# Supplementary material for: Longitudinal Comparison of Constant Artifacts in Optical Coherence Tomography Angiography in Patients with Posterior Uveitis Compared to Healthy Subjects
Source: J Clin Med. 2022 Sep 13;11(18):5376. doi: 10.3390/jcm11185376 (PMC9502304; doi:10.3390/jcm11185376)
Supplement: Supplementary file 1 [file jcm-11-05376-s001.zip › jcm-1860446-supplementary.pdf]

Supplementary Table S1. Artifact types of uveitis group after 3 and 6 months.

	Baseline	3-months	6-months	P - Value
**Artifact 1: system immanent artifacts**				
**Projection**				
SVC	0	0	0	1
SVP	0	0	0	1
DVC	84 (82.35)	84 (82.35)	88 (86.27)	0.169
ICP	85 (83.33)	85 (83.33)	88 (86.27)	0.368
DCP	54 (52.94)	52 (50.98)	52 (50.98)	0.766
CC	0	0	0	1
**Shadowing**				
SVC	7 (6.86)	9 (8.82)	10 (9.8)	0.529
SVP	7 (6.86)	9 (8.82)	10 (9.8)	0.529
DVC	8 (7.84)	12 (11.76)	10 (9.8)	0.449
ICP	7 (6.86)	12 (11.76)	10 (9.8)	0.257
DCP	26 (25.49)	27 (26.47)	28 (27.45)	0.895
CC	44 (43.13)	42 (41.17)	42 (41.17)	0.882
**Window effect**				
SVC	0	0	1	0.368
SVP	0	0	1	0.368
DVC	0	0	1	0.368
ICP	0	0	1	0.368
DCP	0	0	1	0.368
CC	6 (5.88)	8 (7.84)	8 (7.84)	0.264
**Artifact 2: artifacts through image processing**				
**Segmentation**				
SVC	42 (41.18)	43 (42.16)	43 (42.16)	0.882
SVP	42 (41.18)	43 (42.16)	43 (42.16)	0.882
DVC	40 (39.22)	43 (42.16)	43 (42.16)	0.368
ICP	40 (39.22)	43 (42.16)	43 (42.16)	0.368
DCP	23 (22.55)	24 (23.52)	19 (18.63)	0.148
CC	0	3 (4.48)	2 (1.96)	0.097
**Duplication of Vessels**				
SVC	0	0	0	1
SVP	0	0	0	1
DVC	0	0	0	1
ICP	0	0	0	1
DCP	0	0	0	1

CC	0		0	1
**Artifact 3: artifacts through motion**				
**Motion artifact**				
SVC	5 (4.9)	5 (4.9)	3 (2.94)	0.717
SVP	5 (4.9)	5 (4.9)	3 (2.94)	0.717
DVC	1 (0.98)	4 (3.92)	2 (1.96)	0.368
ICP	1 (0.98)	4 (3.92)	2 (1.96)	0.368
DCP	1 (0.98)	3 (2.94)	2 (1.96)	0.607
CC	0	0	1 (0.98)	0.368
**Blink artifact**				
SVC	12 (11.76)	10 (9.8)	15 (14.71)	0.531
SVP	12 (11.76)	10 (9.8)	15 (14.71)	0.531
DVC	14 (13.73)	12 (11.76)	16 (15.69)	0.687
ICP	14 (13.73)	12 (11.76)	16 (15.69)	0.529
DCP	13 (12.75)	12 (11.76)	17 (16.67)	0.529
CC	15 (14.71)	14 (13.73)	20 (19.61)	0.452
**Banding**				
SVC	2 (1.96)	1 (0.98)	1 (0.98)	0.368
SVP	2 (1.96)	1 (0.98)	1 (0.98)	0.368
DVC	2 (1.96)	1 (0.98)	1 (0.98)	0.368
ICP	2 (1.96)	1 (0.98)	1 (0.98)	0.368
DCP	2 (1.96)	1 (0.98)	1 (0.98)	0.368
CC	1 (0.98)	0	0	0.368

CC = choriocapillaris; DVC = deep vascular complex; DCP = deep capillary plexus; ICP = intermediate capillary plexus: SCV= superficial vascular complex; SVP = superficial vascular plexus;
